# Osteoporosis in Healthy South Indian Males and the Influence of Life Style Factors and Vitamin D Status on Bone Mineral Density

**DOI:** 10.1155/2014/723238

**Published:** 2014-11-11

**Authors:** Sahana Shetty, Nitin Kapoor, Dukhabandhu Naik, Hesarghatta Shyamasunder Asha, Suresh Prabu, Nihal Thomas, Mandalam Subramaniam Seshadri, Thomas Vizhalil Paul

**Affiliations:** Department of Endocrinology, Diabetes & Metabolism, Christian Medical College & Hospital, Vellore 632004, India

## Abstract

*Objective*. To study the prevalence of osteoporosis and vitamin D deficiency in healthy men and to explore the influence of various life style factors on bone mineral density (BMD) and also to look at number of subjects warranting treatment. *Methods*. Ambulatory south Indian men aged above 50 were recruited by cluster random sampling. The physical activity, risk factors in the FRAX tool, BMD, vitamin D, and PTH were assessed. The number of people needing treatment was calculated, which included subjects with osteoporosis and osteopenia with 10-year probability of major osteoporotic fracture >20 percent and hip fracture >3 percent in FRAX India. *Results*. A total of 252 men with a mean age of 58 years were studied. The prevalence of osteoporosis and osteopenia at any one site was 20% (50/252) and 58%, respectively. Vitamin D deficiency (<20 ng/dL) was seen in 53%. On multiple logistic regression, BMI (OR 0.3; *P* value = 0.04) and physical activity (OR 0.4; *P* value < 0.001) had protective effect on BMD. Twenty-five percent warranted treatment. *Conclusions*. A significantly large proportion of south Indian men had osteoporosis and vitamin D deficiency. Further interventional studies are needed to look at reduction in end points like fractures in these subjects.

## 1. Introduction

Osteoporosis in men is now recognized as a major underestimated public health problem [1]. With the gradual increase in life expectancy, advancing age related illnesses are increasing [2]. After the age of fifty, one out of three osteoporotic fractures are seen in men. Furthermore, an in depth understanding of this subject has revealed that about fifty percent of these causes are potentially treatable. Studies have shown that men with osteoporotic fractures have a much higher mortality and morbidity when compared to women [3]. This may add on to the economic burden in a developing country like India, where men may be the only earning members in many families [4].

In addition to genetic determinants, several life-style related factors like physical activity, calcium intake, smoking, alcohol consumption, and vitamin D status may influence the bone mass in men [5]. However, the prevalence and influence of these factors may vary according to ethnicity.

Screening for osteoporosis in men is usually recommended above the age of 70 years [1, 5]. However, its relevance in relation to the variability in ethnicity requires validation through prospective studies. There are differences in peak bone mass, body frame, and nutrition and life style factors among various populations [1]. There are no clear guidelines available for screening men with osteoporosis among ethnic groups other than the Caucasian population.

There is also a paucity of data with regards to the risk factors that have been mentioned and their influence on bone health in an Indian context. Many studies have shown that a low Bone Mineral Density (BMD) may not be the sole factor which determines the risk of fracture [6]. This led on to developing a web based tool FRAX (Fracture Risk Assessment) to assess the fracture risk which incorporated various risk factors like parental hip fracture, smoking, alcohol consumption, past history of fracture, and other factors in addition to femoral neck BMD [7]. This tool calculates a ten-year probability of major osteoporotic fracture and hip fracture.

In this study, we have attempted to look at the prevalence of osteoporosis and vitamin D status in healthy South Indian men and to study the influence of various life style factors on bone mineral density. The number of subjects warranting treatment in these healthy subjects was also computed.

## 2. Materials and Methods

The study was a cross sectional one conducted over a duration period of 1 year. This study was approved by Institutional Review Board.

We conducted a survey of the total number of houses in an urban region of south India. Men above 50 years of age in that locality were recruited by cluster random sampling after obtaining a written informed consent. Men with a history of chronic liver or renal disease, hyperthyroidism, hyperparathyroidism, hypogonadism, and malabsorption and those on medications such as anticonvulsants, antiretrovirals, and antituberculous therapy which will affect the bone health were excluded.

The risk factors which were mentioned in the FRAX tool assessment included age, sex, height, weight, a past history of fragility fracture, parental history of hip fracture, history of smoking or alcohol intake (3 or more units/day), the presence of rheumatoid arthritis, and any other history suggestive of secondary osteoporosis [7]. Physical activity was evaluated using a previously published questionnaire standardized for the Indian population and was categorized with a scoring system of less than 1.4 as sedentary, 1.55 to 1.6 as moderately active, and more than 1.75 as strenuous active [8].

An overnight fasting blood sample was obtained for estimation of serum calcium (8.6–10.2 mg/dL), phosphate (2.5–5 mg/dL), albumin (3.5–5.0 gm/dL), alkaline phosphatase (40–125 iu/L), creatinine (0.6–1.2 mg/dL), 25-hydroxy vitamin D (30–75 ng/mL) hereafter referred to as vitamin D, intact parathyroid hormone (iPTH) (8–50 pg/mL), and testosterone (300–1000 ng/dL). These reference ranges are as provided by the local laboratory. Vitamin D deficiency was defined as a vitamin D level of less than 20 ng/mL and a level less than 10 ng/mL was considered to indicate severe vitamin D deficiency [9]. The vitamin D, intact PTH, and testosterone were measured by chemiluminescence method using Immulite analyzer 2000. Other biochemical parameters were measured in a fully automated and computerized microanalyzer (*Hitachi Roche Modular P 800 model*). The intra-assay and interassay coefficients of variation for these analytes were 1 to 5%.

BMD was assessed using the Hologic DXA QDR 4500 Discovery A machine at the lumbar spine and femoral neck by the same technician. Precision was 2 percent at both themeasured sites (spine and neck of femur). The WHO classification was used for categorization of BMD [10]. Osteoporosis was defined as* T* score ≤−2.5, osteopenia, or low bone mass −1 to −2.5 and normal as >−1.

In subjects who were found to have osteopenia, data regarding all risk factors were incorporated into a web based software—Fracture Risk Assessment—FRAX India for calculating the 10-year probability of major osteoporotic fracture and hip fracture. The number of people needing treatment was calculated, which included the total number of subjects with osteoporosis at any site and osteopenia with a 10-year probability of major osteoporotic fracture more than 20 and hip fracture more than 3 as calculated by FRAX India. This was as per the Endocrine Society clinical practice guidelines [11].

### 2.1. Sample Size Calculation and Statistical Analysis

The prevalence of male osteoporosis above the age of 50 years in published literature was about 6% [12]. Total number of men in an urban area of Southern India above the age of 50 years was 2120. The sample size required for a precision of 5% and a 95 confidence interval and a power of 80% was 240. The cluster random sampling was used to recruit subjects for the study. Independent* T*-test was used to compare the means of two continuous variables if they were normally distributed and nonparametric tests were used if their distribution were not normal. Statistical analysis was done using the SPSSv.16.0 software (IBM Corp., USA).

## 3. Results 

Two hundred and fifty-two men above the age of 50 years were included in the study. The demographic parameters, biochemistry including vitamin D status, and BMD of the study subjects are summarized in Table 1.

The mean age (SD) of the study subjects was 58 (11.8) years and the mean (SD) BMI was 23.3 (4.5) kg/m^2^. Forty percent (*n* = 100) of men were above the age of 60 years. In relation to physical activity, 77 percent were sedentary with the rest being moderately to strenuously active.

**Figure 1:**
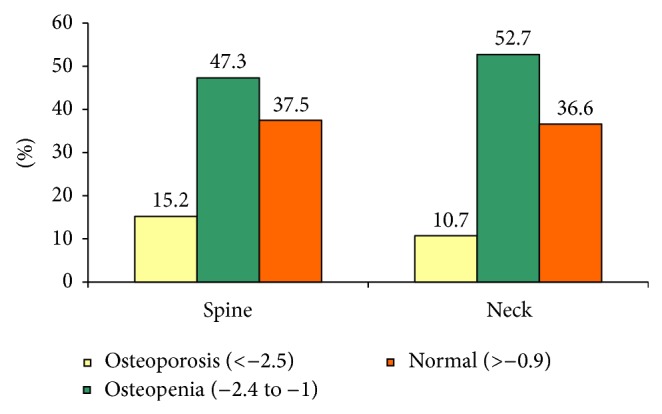
Showing categorization of BMD in the study subjects.

The prevalence of osteoporosis and osteopenia at spine and femoral neck is shown in Figure 1. The osteoporosis was seen in 15% and 10% of the subjects at the spine and femoral neck, respectively. A significantly greater proportion of subjects aged above 60 years had osteoporosis at the femoral neck when compared to men below that age (15.6% versus 7.5%, *P* = 0.01). Osteoporosis at the spine was also more commonly seen among subjects aged above the age of 60 years; however, it was not statistically significant.

Overall the prevalence of osteoporosis and osteopenia at any one site was 20% (50/252) and 58% (146/252), respectively. However, in men aged above 60 years, the prevalence of osteoporosis was 45%. Vitamin D deficiency (<20 ng/dL) was seen in 53% (*n* = 133) and 7% had severe vitamin D deficiency (<10 ng/dL). Eight percent of subjects had biochemical hypogonadism (<300 ng/dL).

Among the risk factors studied, BMD at the femoral neck was significantly higher in subjects who were physically more active when compared to men who had a sedentary lifestyle (0.811 versus 0.733 gm/cm^2^, *P* = 0.005). Multiple logistic regression analysis was performed to assess the impact of various factors that influence bone mineral density at femoral neck in healthy male subjects. These included body mass index, hypogonadism, age, vitamin D deficiency, smoking, alcohol use, and physical activity. We found that body mass index (OR 0.3; CI 0.1 to 0.40;* P* value = 0.04) and physical activity (OR 0.4; CI 0.12 to 0.9;* P* value < 0.0001) had a statistically significant protective effect on bone mineral density.

Amongst the subjects who were having osteopenia at any one site (*n* = 146) 13 men had a greater than 3% probable risk of hip fracture; however, none of these subjects had more than a 20 percent risk of major osteoporotic fracture calculated by FRAX India.

Therefore, out of the total 256 subjects, 63 warranted treatment (50 had osteoporosis plus 13 osteopenics with a more than 3% probable risk of hip fracture). The mean (SD) age of subjects warranting therapy in our study was 67.5 (11.65) years. There was a significant negative correlation between vitamin D and PTH levels (*r* = −0.29, *P* = 0.04). However, there is no significant correlation between vitamin D and BMD at any site and also no significant correlation was found between PTH and BMD.

## 4. Discussion 

Male osteoporosis is an underreported public health problem. In our study, we attempted to look at the prevalence of osteoporosis and the various risk factors in South Indian healthy men above the age of 50 years.

In this present study, about one-fifth of them had osteoporosis at any one site. Vitamin D deficiency was found in over half of the study population. Men who were physically active or having higher body mass index had a better BMD at femoral neck. As per the FRAX score, about 10 percent of the osteopenic subjects had more than a 3%, ten-year probability of sustaining a hip fracture. Mean age of warranting treatment to prevent fractures was close to the recommendations published in the international guidelines [11]. Treatment was warranted in one out of four healthy subjects who had either osteoporosis or osteopenia with a computed ten-year probability of hip fracture of more than 3% as per the FRAX score.

### 4.1. Prevalence of Osteoporosis

In previously published literature, a 9 percent prevalence of osteoporosis has been reported in Northern India [13] and in oriental men [14]. However, in another study at Rochester [15], a 19% prevalence of osteoporosis has been reported, which bears similarity to our study. The differences in the prevalence that was seen between south Indian and north Indian subjects would have been due to many factors like genetic, nutritional, and other environmental factors. They need to be looked at in further prospective studies.

BMD begins to decline after the third decade and is influenced by genetic and environmental factors. Prepubertal BMD is similar in both sexes. However, the pubertal increase in BMD is more in men when compared to women due to a greater cross sectional area in view of increased periosteal apposition under the influence of androgens [16]. Bone remodeling with aging leads to trabecular thinning in men, whereas trabecular connectivity is lost in women [17]. Decline in BMD in men may begin as early as 30–40 years. However, the accelerated menopausal bone loss may not be seen. The estimated rate of bone loss with aging in men is about 1% per year [16].

### 4.2. Osteoporosis and Risk of Fracture: Morbidity, Mortality, and Economic Burden Associated with Fracture

The most dreaded complication of osteoporosis is hip fracture, which has been reported to be more in men when compared to women [18]. Osteoporosis in men accounts for more than 30–40% of overall fracture. Fracture in men follows a bimodal presentation with peaks at adolescence and after 60 years [19]. The morbidity and mortality associated with hip fractures in men has been reported as high as 33% [18, 20]. Moreover, a three to four times higher mortality rate has been described in men with hip fracture when compared to females [3, 21].

The economic burden of osteoporotic fractures is not only borne by the patient but by the country as a whole [22, 23]. The major costs may not only include that of investigations, surgery, or long term management of these patients but also the large number of man hours that is lost by these men after they sustain a fracture. This is particularly relevant in developing countries like India where there is an increase in the aging population [24]. About 25% of our subjects needed treatment in accordance with endocrine society guidelines [11]. Considering the population of men above 65 years in India (about 27 million) and the available incidence of hip fracture in men above 50 years (105/100,000), the magnitude of the problem is huge [25, 26]. It translates into having about 25,000 hip fractures in elderly men (>65 years) every year and about one-third to one-fourth of them dying in 1 to 2 years. The mere cost of treating each fracture is about 90,000 rupees (about 1500 US dollars) in India showing huge economic burden associated with them. At this point, there is a limited availability of DXA scanners in the country excluding major cities [24]. So, the health care system at the primary level can use FRAX scoring (utilizing BMI without BMD) to plan preventive strategies which in turn will decrease the morbidity, mortality, and economic costs associated with them. However, further studies are needed with such interventions to look at the impact of these in a larger cohort of elderly men. It will enable the national bodies to come up with clinical practice guidelines relevant in an Indian context.

### 4.3. Vitamin D Deficiency: Its Impact on Bone Health

A high prevalence of vitamin D deficiency has been reported in various cohorts of Indian population [27]. Vitamin D deficiency can either decrease mineralization or cause secondary hyperparathyroidism or both resulting in a low bone mineral density [28]. A low vitamin D may also cause proximal myopathy predisposing these patients to a fall and subsequent fracture [27].

### 4.4. Role of Physical Activity and BMI

A positive impact of physical activity on bone mass or BMD demonstrated in our study is probably due to a skeletal response to mechanical strain by stimulating bone formation [29]. Also increase in the muscle strength and neuromuscular function seen secondary to physical activity has been shown to decrease the incidences of fall and a resulting fracture [30]. A reduction in fracture risk has also been reported on follow-up in subjects who were physically active [31].

An increase in body mass index had a significant impact on BMD. Several studies have shown the positive correlation between BMI and BMD. This is explained by a higher gravitational load on the femur neck, increased peak bone mass, and higher circulating estradiol level [28].

## 5. Limitations of the Study

The socioeconomic status, nutritional status, prevalent vertebral fractures, and bone turnover markers in these individuals were not assessed. Also this data has been collected from an urban society in southern India and cannot be generalized to the entire Indian population.

## 6. Conclusions

A significantly larger proportion of otherwise normal healthy men in our community had osteoporosis and vitamin D deficiency compared to previously published studies. Men with a higher BMI were physically active and had a better BMD. Large scale prospective studies with interventions are needed to look at the reduction in the end points like number of incident fractures and morbidity associated with them.

## Figures and Tables

**Table 1:** Demography, biochemistry, and BMD.

Variables	Mean (standard deviation)	Range
Age (years)	58.8 (11.8)	51–74
Height (cms)	165.4 (7.2)	142.2–183.0
Weight (kgs)	63.7 (14.2)	35–114
BMI (kg/m^2^)	23.3 (4.5)	16.2–41.6
Corrected calcium (mg%)	8.82 (0.43)	8.1–10.2
Phosphorus (mg%)	3.9 (0.5)	2.1–5.1
Creatinine (mg%)	0.96 (0.12)	0.7–1.3
Vitamin D (ng/mL)	20.4 (8.3)	4–58
Alkaline phosphatase (U/L)	73.5 (21.4)	39–167
Testosterone (ng/dL)	620 (124)	270–980
PTH (pg/mL)	44.5 (25.6)	14.5–151.0
BMD spine (gm/cm^2^)	0.943 (0.111)	0.912–0.974
BMD neck (gm/cm^2^)	0.761 (0.124)	0.733–0.771
